# Ketamine, Psychedelics, and Psychotherapy: Reframing, Redefining, Renaming Treatment Models

**DOI:** 10.1177/07067437251389090

**Published:** 2025-10-28

**Authors:** Jennifer Swainson, Elisa Brietzke, Atul Khullar, Roger S. McIntyre, Claudio N. Soares

**Affiliations:** 1 Department of Psychiatry, 3158 University of Alberta, Edmonton, Canada; 2 Neuroscience and Mental Health Institute, 3158 University of Alberta, Edmonton, Canada; 3 Department of Psychiatry, 4257 Queen's University School of Medicine, Kingston, ON, Canada; 4 Centre for Psychedelics Health and Research, Providence Care Hospital and Queen's University, Kingston, ON, Canada; 5 Department of Psychiatry, 654596 University of Toronto, Toronto, Canada; 6 Department of Pharmacology, University of Toronto, Toronto, Canada

**Keywords:** psychedelics, ketamine, psilocybin, MDMA, nomenclature, terminology, depression, PTSD, psychotherapy, therapy

## Abstract

There has been a renewed interest in the use of various psychedelic agents as potential therapies for multiple psychiatric conditions, including post-traumatic stress disorder (PTSD), major depressive disorder (MDD), generalized anxiety disorder (GAD), to name a few. This follows the recent accumulation of evidence for ketamine pharmacotherapy and a rapid proliferation of clinics/programs offering a variety of ketamine based treatments. A quick glance at the existing evidence, however, reveals a confusing scenario for patients, healthcare providers, and regulators. Overall, there are no standard definitions of what constitutes a psychotherapeutic intervention within a psychedelic-based or a ketamine-based treatment. More specifically, studies have not always distinguished between using a well-known, manualized psychotherapy, providing psychoeducation and psychological support, or providing a therapy specifically to integrate the drug experience in psychedelic trials. Also, it is difficult to determine the role of the psychedelic agent as a stand-alone treatment, and the relative importance (if any) of the psychedelic experience for the desired therapeutic effect. In this perspective, we discuss the evolving landscape of psychedelic-based and ketamine-based treatments, highlighting different therapeutic models, their methodologies, and the need for clearer definitions and rigorous clinical trials. The document proposes three new definitions to improve clarity in evaluating the effects of these agents and the role of psychotherapies. We suggest language that will distinguish: (1) when the drug is used for its pharmacologic effects as a stand-alone treatment, without requiring the psychedelic experience or combined psychotherapy; (2) when the treatment requires the acute psychological effects of the drug to assist psychotherapy and (3) When ketamine or a psychedelic agent is used in combination with a structured, manualized psychotherapy that could be implemented even in the absence of these agents. We hope that this new terminology and definitions will help distinguish the various therapeutic roles of these agents (as stand-alone treatments or in combination with psychotherapies), and facilitate study designs, regulatory pathways, and more informed patient care.

## Plain Language Summary Title

Ketamine, Psychedelics, and Psychotherapy: Understanding treatment models to better inform practice

Plain Language Summary

The use of psychedelics to improve symptoms of post-traumatic stress disorder (PTSD), major depressive disorder (MDD), and generalized anxiety disorder (GAD has gained significant attention lately, following the rapid proliferation of clinics/programs offering ketamine-based therapies.

However, it can be confusing for patients, healthcare workers, and regulators to understand what all these treatments actually involve. There are no standard definitions of what a psychotherapeutic intervention involving psychedelics or ketamine truly means.

More specifically, studies often haven't clearly separated the use of well-established, manualized psychotherapies from simply offering psychological support or psychoeducation during psychedelic trials. It can also be challenging to understand the role of the psychedelic itself as a standalone treatment, as well as how much the psychedelic experience contributes to the positive therapeutic outcomes, if at all.

Here, we explore the changing world of psychedelic- and ketamine-based treatments, highlighting various therapeutic approaches, their methods, and the importance of clearer definitions and thorough clinical trials. The document introduces three new definitions to help clarify how these agents are evaluated and their relationship with psychotherapies. We suggest language that clearly distinguishes: 1) when the drug is used for its pharmacologic effects alone, without needing the psychedelic experience or combined therapy; 2) when the drug's immediate psychological effects are used to support therapy; and 3) when ketamine or another psychedelic is used alongside a structured, manualized psychotherapy that can be offered even without these agents.

We hope that this new terminology and definitions will help distinguish the various therapeutic roles of these agents (as stand-alone treatments or in combination with psychotherapies), and facilitate study designs, regulatory pathways, and more informed patient care.

Psychedelic Assisted Psychotherapy (PAP) has captured interest not only in psychiatry, but also among the public and layperson media. Much hype surrounded the application for 3, 4 Methylenedoixymethamphetamine (MDMA) to the United States Food and Drug Administration (USFDA) as a treatment for post-traumatic stress disorder (PTSD). MDMA was studied in protocols using MDMA-assisted psychotherapy (MDMA-AP), where the intervention was a psychotherapy focused on integrating the psychedelic experience to facilitate processing of previous trauma. The potential for a mystical and transformative experience to alleviate suffering brought widespread appeal, given that traditional treatments take time and are not always effective. The positive outcomes of the MDMA trials^1^ prompted interest in MDMA-AP for PTSD and PAP with other psychedelic drugs, for other mental health conditions.

The USFDA ultimately rejected MDMA-AP due to various methodologic concerns with the studies, including concern that study design made it impossible to determine the effects of the drug itself and to distinguish it from the potential role of the psychedelic ‘trip’, or the role of the psychotherapy. A recent Editorial^2^ addressed this concern on a broader level, pointing out that when using psychedelics for mental health conditions, the potential pharmacologic benefits or harms of the drugs themselves are still unclear, and their use is not without potential risks. The psychedelic experience has not been established as necessary for the therapeutic benefits, and the potential psychological harms associated with this process have also not been fully evaluated. The authors^2^ called for improved rigour in clinical trials, suggesting randomized groups undergoing psychotherapy alone, psychedelic drug alone, and the combination of psychotherapy and psychedelic drugs.

While an improved study design could support establishing the role of the drug itself, the pathway to determine the role of psychotherapy is less clear.^2^ Psychotherapy is a broad term, with many interpretations and potential approaches. The combination of psychoeducation, support, and/or manualized therapies with various traditional pharmacotherapies has been demonstrated as superior to the use of pharmacotherapy alone for several mental health conditions. But, if the pharmacotherapy includes a psychedelic agent, there exists a widespread assumption that psychotherapy would not be an established manualized therapy, but a new process that centres therapy around the ‘trip’ itself. This ‘psychedelic therapy model’ is based mainly on the early work of a psychedelic pioneer, Stanislav Grof, who worked on the premise that a psychedelic (high) dose of the drug would induce a transformational experience, thought to facilitate an opening of the unconscious to otherwise inaccessible psychological material.^3^ This model of therapy is typically structured with three components: preparation for the psychedelic experience, medication administration (dosing session) and integration.^4^ Therapy may or may not take place while the patient is under the acute effects of the drug. Integration sessions are held after the dosing sessions to process the psychedelic experience, and this may occur on the same day or on a different day. Another model uses lower ‘psycholytic doses’, which are thought to relax psychological defences to enhance the therapy process,^5^ without leading to a full psychedelic experience. In this case, therapy is conducted after the drug is ingested.

While the above models require the acute psychological effects of the drug, others take a ‘plasticity-oriented approach’, proposing that neuroplastic effects of these agents^6^ could enhance outcomes of other structured psychotherapies. For example, in the Yale model of PAP using psilocybin for depression, the therapy follows an acceptance and commitment therapy (ACT) model, rather than an integration of the psychedelic experience itself.^7^ Similarly, in a systematic review of psilocybin for substance use disorders (SUD), most studies with a psychotherapy intervention included established therapies such as motivational enhancement (ME) or cognitive behavioural therapy (CBT).^8^ These studies applied manualized therapies – already known to improve outcomes when combined with other appropriate pharmacotherapies.

In effect, three very different treatment models could be delineated to accurately evaluate and describe the effects of psychedelics, the psychedelic experience, and the role of psychotherapy. A reframing of psychedelic models could consider (1) Psychedelic Pharmacotherapy (PP): a model where the drug is used for its pharmacologic or neuroplastic effects as a stand-alone treatment. In this case, the non-pharmacological aspect of the intervention is solely focused on establishing a safe space for the drug administration and patient experience, (2) Psychedelic Assisted Psychotherapy (PAP): a treatment model that requires the acute psychological effects of the drug (whether psychedelic or psycholytic) with the use of a psychotherapeutic intervention that is focused on integration of the experience, and (3) Psychedelics Combined with Psychotherapy (PCP): where, like the Yale model, a psychedelic agent is used for its presumed neuroplastic effects in combination with a known structured or manualized therapy – one that could be effective on its own.

Distinguishing among treatment models becomes even more important when considering ketamine-based treatments, which, unlike psychedelics, have more established evidence for their use as stand-alone agents, are more widely available, and are increasingly used to support psychotherapy through PAP and PCP models. Though not a classic psychedelic agent, ketamine is used as an antidepressant and may cause a dissociative experience, which is considered a treatment-emergent adverse event when ketamine is used as pharmacotherapy. This dissociative experience is often characterized by dizziness, sensory sensitivity, misperceptions or illusions, derealization and depersonalization. This premise is central to one model of Ketamine Assisted Psychotherapy (KAP), where the dissociation and a separation of mind from body are considered therapeutic, supporting an ‘openness’ to new ways of thinking, while also allowing a ‘time out’ from the usual mind and from negativity.^9^ It is a desired and necessary part of the psychotherapy. Consistent with the model adopted for PAP, this form of KAP typically involves a preparatory session, the administration of medication (which may or may not include therapy during dissociation), and one or more integration sessions afterwards to process the experience. Clinics offering this form of treatment often promote (with varying degrees of evidence) ketamine's rapid antidepressant effects and its potential benefit for PTSD and multiple other psychiatric conditions, such as SUD, eating disorders, anxiety disorders, and obsessive-compulsive disorder (OCD). As with psychedelics, it is important to assess the therapeutic roles of both the drug and the psychotherapy. For some indications, data clearly support the benefit of ketamine in the absence of psychotherapy. Both the Canadian Network for Mood and Anxiety Treatments (CANMAT) 2023 Depression treatment guidelines^10^ and CANMAT 2018 Bipolar treatment guidelines^11^ recognize the evidence for ketamine pharmacotherapy (KP) treatment for depression and bipolar depression. Systematic reviews also suggest emerging evidence for ketamine as pharmacotherapy treat PTSD, Treatment-Resistant PTSD,^12^ OCD,^12^ at-risk drinking,^12^ alcohol use disorder,^12,13^ and cocaine use disorder.^12,13^

This existing pharmacotherapy data provides a groundwork for inquiry into the role of therapy and demonstrates that not all patients need therapy to respond to ketamine. However, benefits from an acute course of KP are not typically sustained beyond a few weeks, and strategies such as maintenance ketamine^14–16^ or combining the ketamine with psychotherapy^17^ may extend the ketamine response. While both models require further research to determine the most effective approaches, the evaluation of psychotherapy methods has been hindered by unclear definitions and terms for the so-called Ketamine-Assisted Psychotherapy. This term has also been referred to as Ketamine-Assisted Therapy or simply ‘ketamine therapy’, which may also sometimes be used in reference to intravenous ketamine administered as a pharmacotherapy, confusing the ketamine landscape even further.

Like with PAP/PCP, it is imperative to reframe treatment models and to develop consistent terms. The redefined models proposed earlier in this perspective may also be applied to ketamine renaming models as: (1) Ketamine Pharmacotherapy (KP) where ketamine is administered at any dose via any route for its direct pharmacologic effect; (2) Ketamine Combined with Psychotherapy (KCP) which *combines* ketamine with a structured, manualized form of psychotherapy; and (3) Ketamine Assisted Psychotherapy (KAP), which requires ketamine's acute psychological effects as a focus of therapy to *assist* the psychotherapy.

The adoption of these terms will facilitate a more accurate evaluation of existing literature and future studies. Current literature has a broad definition, including any use of psychotherapies with ketamine. Failure to distinguish between approaches risks inaccurate claims for the safety and efficacy of various interventions. For example, a 2022 systematic review^17^ of ‘KAP’ for psychiatric indications included three studies on patients with depression, all of which suggested positive results. Consequently, patients and clinicians may conclude that the dissociative experience and its integration could be beneficial for treating depression. However, only one of the three studies adopted this approach, and it was a case report involving only two patients with comorbid major depressive disorder and an eating disorder. The more robust data came from the two other studies, which, by our definitions, would not be categorized as KAP but rather as KCP. An open-label trial and a randomized controlled trial included a course of CBT in conjunction with IV ketamine. Those who received CBT experienced a longer duration of response compared to those without CBT – in this case, the psychotherapy appeared to enhance the response to pharmacotherapy.

Considering that both ketamine and CBT separately have strong efficacy data for depression, a positive outcome with the combined intervention is unsurprising. Moreover, it is known that for treatment-resistant or moderate to severe depression, combining pharmacotherapy and psychotherapy can lead to better outcomes.^10^ CBT is a proven therapy that could be done alone or with any medication – in this case, it just happens to be ketamine.

Further examples reinforce the need to distinguish between the KAP and KCP approaches. In a systematic review^13^ of ketamine for SUD, several studies included psychotherapies and were therefore categorized as KAP. But, on closer look, most psychotherapies included in the studies actually used a KCP approach with existing manualized therapies – that is, motivational enhancement therapy (MET) for alcohol use disorder,^13^ mindfulness-based relapse prevention (MBRP) for cocaine use disorder,^13^ and both MET and MBRP for cannabis use disorder.^13^ Considering our redefined terms, evidence to support a KAP approach would be limited to two studies with in patients with opiate use disorder.^13^ Similarly, in a recent systematic review of novel PTSD treatments, reports of combining ketamine with psychotherapy also generally used a manualized therapy such as exposure therapy or cognitive processing,^18^ once again consistent with a KCP model. So, is the dissociative experience and its integration necessary? Current terminology fails to provide a clearly define this question and may cause confusion for both patients and healthcare providers.

Other challenges arise in the consideration of interventions or procedures described as ‘psychological support’, a phrase commonly encountered in the psychedelic literature. While this is an intervention, we suggest that this not be considered a form of psychotherapy, but a necessary procedure when administering a drug that is likely to elicit a dissociative or psychedelic effect. The KP/PP models should include psychoeducation about potential drug effects, an essential step for informed consent, both in research and clinical care. Additionally, if a patient or research participant becomes distressed, reassurance and support must be provided. It is essential, moving forward, to clarify whether ‘support’ is limited to pre-education and safety assistance during the dosing sessions or includes engagement in follow-up sessions to discuss the experience. If the latter, this resembles an integration session more aligned with a KAP/PAP model.

Terminology also becomes important from a regulatory standpoint. As the practice of KAP and interest in PAP have gained popularity, regulations surrounding these practices are evolving. In order to protect patients receiving psychotherapy while in a vulnerable mental state, regulatory bodies are starting to require facilities offering PAP to undergo accreditation processes and meet their standards. For example, under the Alberta Mental Health Services Protection Act (AMHSPA),^19^ ketamine is listed as a psychedelic agent along with psilocybin, psilocin, MDMA, LSD, mescaline (peyote), DMT, and 5-methoxy-DMT. As such, KAP practices would fall under the umbrella of PAP. The Act broadly defines PAP as ‘services to treat a psychiatric disorder with psychotherapy and one or more designated psychedelic drugs, whether or not the administration of the drug and the psychotherapy are provided on the same day or on different days’.

Without properly defined terms, such regulations may have unintended consequences and restrict access to care. The Alberta legislation exempts ketamine when prescribed as a pharmacotherapy – that is, our definition of KP. The legislation aims to ensure that KAP/PAP is delivered with adequate consideration for the physical and psychological safety of patients. But, by current definitions, a patient doing CBT alongside KP would fall under the category of KAP. Placing similar accreditation requirements for the use of established manualized therapies in patients who are treated with KP is unnecessary and may limit access. Using our terms, this is a KCP model which does not carry the same psychological vulnerability that may be associated with KAP. Regulation and protection of patients are paramount as this field evolves rapidly. Still, we must employ the appropriate language to accurately distinguish what needs to be regulated before addressing how it should be regulated.

The use of these new terms will also enhance the clarity of clinical trial design, facilitate the evaluation of the literature, and aid in educating patients. While KAP and KCP, as we have defined them, remain broad, this language will support better evaluation and understanding of the role of the dissociative or psychedelic experience in the treatment of various indications. It should be noted that both KAP and KCP practices may involve single versus multiple doses of ketamine, variable dosing strengths or modes of administration, and a variable number of therapy sessions, which may be done individually or in a group. Further, the timing of therapy sessions and medication administration is heterogeneous. Both the ketamine itself and the therapy may be considered in the acute treatment phase, or as maintenance treatments.

While this perspective was not a comprehensive evaluation of the evidence, it clearly demonstrates that language recently in use may, at times, be misleading to both patients and healthcare providers. In particular, there is a risk of overstating the evidence base for KAP/PAP, which remains largely experimental, and is currently recommended only for individuals who have not responded to more evidence-based treatments.^20^ Accurate reporting of not only risks but also evidence-based information is necessary for clinicians to obtain and for patients to provide informed consent for treatment.

As the field moves forward, we encourage researchers, clinicians, and regulatory bodies to adopt this terminology. This will support more transparent reporting and trial design, providing a language for regulatory bodies to consider when addressing patient safety. While these proposed models introduce a novel and essential framework for categorizing interventions, the heterogeneous nature of ketamine and psychedelic practices also requires additional details concerning both the drug and the therapy used. Future reporting should include the classification of treatment model, the dose (range) and route of administration of drug used, and whether it is expected to elicit psychedelic or psycholytic effects. Once categorized as KAP or KCP, model used should be more clearly desscribed used with consideration for timing, number of sessions, and model.

As clinicians and researchers continue to pursue answers to so many unanswered questions in this field,^21^ we hope the proposed framework and terminology will guide critical thinking and review of the existing literature while informing better design and implementation of future studies.

Table 1 provides a summary of our suggested reframing, redefining, and renaming of these treatment models.

**Table 1.** Proposed Ketamine and Psychedelic Treatment Models.

Ketamine treatment models	
**Ketamine Pharmacotherapy (KP)** Any dose	Administration of ketamine via any route, for the purpose of its pharmacologic effects. Any dissociative experience is considered a treatment emergent adverse event and not necessary for efficacy.
	As required, support is given during the experience to avoid or minimize patient distress.
**Ketamine Combined with Psychotherapy (KCP)** Dissociative doses (higher doses likely to cause acute dissociative side effects) or Microdoses (lower doses not expected to typically cause dissociative effects)	Ketamine Pharmacotherapy, with a psychotherapy as usual in parallel. The therapy could be done with or without any medication and is not specific to any qualities of ketamine. Neuroplastic effects of the drug are thought to enhance the effects of therapy.
	Therapy is done before or after ketamine dosing, on same day or, more frequently, on different days.
**Ketamine Assisted Psychotherapy (KAP)** Dissociative Doses (higher doses likely to cause acute dissociative effects) or Psycholytic dose (lower doses expected to elicit a relaxing of defenses)	Administration of Ketamine is primarily for experience of dissociative or psycholytic effects. Psychotherapy may be informed by various psychotherapeutic models/approaches and typically involves preparation for, and integration of the dosing experience. Pharmacologic benefits may also occur.
	Therapy may be done before, during or after ketamine experience, on the same or different days.
**Psychedelics Treatment Models**	
**Psychedelic Pharmacotherapy (PP)** Any dose	Administration of a psychedelic at any dose, via any route, for the purpose of its pharmacologic effects. If a psychedelic experience occurs, it is considered a treatment emergent adverse event and not necessary for efficacy.
	As required, support is given during the experience to avoid or minimize patient distress.
**Psychedelic Combined with Psychotherapy (PCP)** Psychedelic dose (higher doses expected to elicit acute psychedelic effects) Psycholytic dose (lower doses expected to elicit a relaxing of defences) or Microdose (Very low doses not expected to elicit psychedelic effects, or psycholytic effects)	Psychedelic Pharmacotherapy, with a psychotherapy as usual in parallel. The therapy could be done with or without any medication and is not specific to any qualities of the psychedelic drug. Neuroplastic effects of the drug may be thought to enhance the effects of therapy.
	Therapy is done before and/or after administration of the psychedelic. Psychotherapy is done after drug effects have dissipated, and most frequently occurs on a different day than psychedelic dosing.
**Psychedelic Assisted Psychotherapy (PAP)** Psychedelic dose (higher doses expected to elicit acute psychedelic effects) Psycholytic dose (lower doses expected to elicit a relaxing of defenses)	Administration of a psychedelic drug with aims to elicit acute psychological effects. Psychotherapy may be informed by various models/approaches, and typically involves preparation for, and integration of the dosing experience. Pharmacologic benefits may also occur.
	Therapy may be done before, during or after the experience, on the same or different days.
